# Macrophage-derived chemokines in T cell regulation: implications for cancer immunotherapy

**DOI:** 10.3389/fimmu.2025.1739154

**Published:** 2026-01-19

**Authors:** Kunpeng Zhang, Jingjing Liu, Qi Liu, Ningning Zhu, Baodong Ye

**Affiliations:** 1 Department of Hematology, First Affiliated Hospital, Zhejiang Chinese Medical University, Hangzhou, China; 2 The First School of Clinical Medicine, Zhejiang Chinese Medical University, Zhejiang, China; 3 Laboratory of Integrated Traditional Chinese and Western Medicine for Hematology, The First Affiliated Hospital of Zhejiang Chinese Medical University, Zhejiang, China

**Keywords:** cancer immunotherapy, chemokines, macrophages, T cells, tumor microenvironment

## Abstract

Macrophages are pivotal regulators of immunity, with intercellular communication being a central mechanism of their function. Among these communications, chemokines act as critical messengers in macrophage-T cell crosstalk. This review systematically elucidates the notable roles of macrophage-derived chemokines in modulating T cell homeostasis, particularly concentrating on their influence on both CD4^+^ and CD8^+^ T cell differentiation, proliferation, exhaustion, secretory activity, metabolic reprogramming (involving glycolysis and OXPHOS), chemotaxis, and memory formation. In the tumor microenvironment (TME), the dualistic nature of chemokines was highlighted: tumor-associated macrophages (TAMs) could secrete immunosuppressive factors, such as CCL22 and CCL5, recruiting inhibitory cells and inducing CD8^+^ T cell exhaustion. In contrast, M1-like macrophages could produce CXCL9 and CXCL10, activating effector CD8^+^ T cells, thereby enhancing anti-tumor immunity. Finally, the promising therapeutic potential of targeting specific chemokine signaling axes, such as CCL2/CCR2 and CXCL10/CXCR3, was discussed as a strategy to improve the efficacy of cancer immunotherapy.

## 1 Introduction

Chemokines represent a family of signaling proteins that are indispensable for coordinating immune cell migration, activation, and intercellular communication. These functions are critical for maintaining physiological immunity and are also intimately involved in the pathogenesis of various diseases (1). As prolific sources of these signals, macrophages emerge as pivotal regulators of T cell biology. Their secretion of a diverse array of chemokines allows them to modulate multiple facets of T cell activity, including development, differentiation, metabolic reprogramming, migration, and effector functions (2–6). This regulatory role is particularly consequential within the tumor microenvironment (TME), where the dysregulated expression of chemokines by tumor-associated macrophages (TAMs) is a well-established driver of immune evasion and tumor progression (5).

Consequently, this review aimed to systematically elucidate the mechanisms through which macrophage-derived chemokines govern T cell homeostasis and immune function. This review advances beyond previous reviews by systematically addressing the multifaceted roles of macrophage-derived chemokines in modulating T cell homeostasis, particularly concentrating on their influence on metabolic reprogramming, including glycolysis and oxidative phosphorylation (OXPHOS). In contrast to earlier reviews that primarily concentrated on immune activation or suppression, this review explored the dualistic nature of chemokines in the TME, emphasizing their context-dependent roles in promoting both anti-tumor immunity and immune evasion. Additionally, it provided a comprehensive analysis of specific chemokine signaling axes, such as CCL2/CCR2 and CXCL10/CXCR3, and their therapeutic potential in cancer immunotherapy. By integrating these novel insights, this review provided a more complete understanding of the complex interaction between macrophages and T cells, highlighting emerging strategies for potentiating T cell-mediated anti-tumor responses through targeted chemokine modulation.

## 2 Macrophage origin and plasticity

Macrophages originate from embryonic precursors or are derived from bone marrow monocytes (7, 8). Their defining characteristic is remarkable functional plasticity, enabling them to adapt to diverse microenvironments and meet varying immunological demands. Historically, macrophages were broadly categorized into two primary phenotypes: the classically activated phenotype (often termed M1-like), which is pro-inflammatory, and the alternatively activated phenotype (often termed M2-like), associated with anti-inflammatory and tissue-repair properties (9). This classification was originally based on the helper T cell (Th)1/Th2 paradigm and reflected the dual roles of macrophages in both the initiation and resolution of inflammation. However, it is currently understood that macrophage phenotypes are not fixed while rather exist along a dynamic spectrum, continuously regulated by local microenvironmental cues.

Although initial models simplified macrophage activation into two major types, including pro-inflammatory M1-like and anti-inflammatory M2-like, it is widely recognized that macrophages exhibit a remarkable functional diversity. Their phenotypic state can be shaped by a range of stimuli from the surrounding microenvironment. For instance, interferon-γ (IFN-γ) alone or in combination with lipopolysaccharide promotes a pro-inflammatory, M1-like macrophage phenotype, while interleukin (IL)-4 and IL-13 drive polarization toward an anti-inflammatory, M2-like profile (10, 11). In the TME, for instance, TAMs mainly exhibit functional properties resembling the M2-like phenotype and may contribute to immune evasion and disease progression (5).

Functionally, macrophages with an M1-like orientation are adept at phagocytosing pathogens, clearing damaged cells, and presenting antigens, which are crucial for host defense (12). In contrast, those with an M2-like orientation are specialized in tissue remodeling and repair, typically displaying reduced antigen-presentation capacity (13). Beyond these primary functions, macrophages are key modulators of adaptive immune responses through the secretion of chemokines, playing a pivotal role in regulating T cell functions and overall immune responses (14).

## 3 Chemokine structure and function

**Figure 1:**
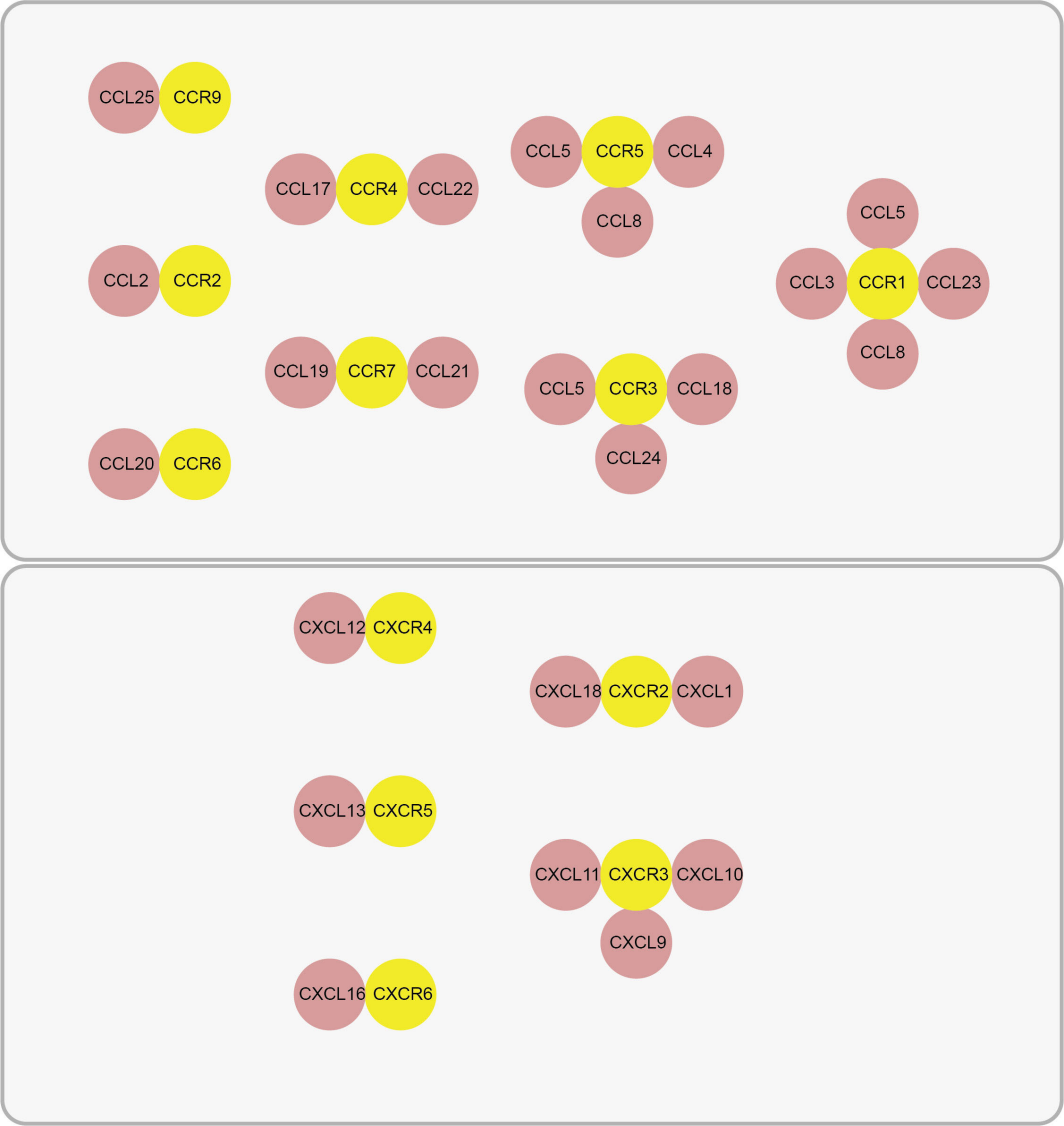
Chemokines and their receptors. CCR9 Ligand (CCL25); CCR2 Ligand (CCL2); CCR6 Ligand (CCL20); CCR4 Ligand (CCL17, CCL22); CCR7 Ligand (CCL19, CCL21); CCR5 Ligand (CCL4, CCL5, CCL8); CCR3 Ligand (CCL5, CCL18, CCL24); CCR1 Ligand (CCL3, CCL5, CCL8, CCL23). CXCR4 Ligand (CXCL12); CXCR5 Ligand (CXCL13); CXCR6 Ligand (CXCL16); CXCR2 Ligand (CXCL1, CXCL18); CXCR3 Ligand (CXCL9, CXCL10, CXCL11).

Chemokines are a family of low-molecular-weight (8–10 kDa) signaling proteins characterized by their short half-lives (15). Their classification into four distinct subfamilies (CC, CXC, XC, and CX3C) is defined by the arrangement of conserved N-terminal cysteine residues. A key structural determinant is the presence or absence of an intervening amino acid between the first two cysteines; this motif critically influences the protein’s tertiary structure, stability, and receptor binding affinity (15). The primary biological function of chemokines is executed through their binding to specific G-protein-coupled receptors (GPCRs), as shown in **Figure 1**, which illustrates the receptor-ligand interaction and the resulting signaling cascade. This ligand-receptor interaction initiates intracellular signaling cascades that govern essential leukocyte behaviors, including directed migration, survival, and functional polarization (16–18). Consequently, chemokines are fundamental orchestrators of immune cell trafficking, activation, and communication, thereby coordinating the responses of lymphocytes (B and T cells) and innate immune cells like natural killer (NK) cells (19–21). This precise coordination is indispensable for launching effective antimicrobial defenses and for maintaining immune homeostasis (22). However, pathogenic dysregulation of chemokine signaling disrupts this equilibrium and contributes to a spectrum of diseases, ranging from chronic inflammatory and autoimmune disorders to cancer (18). For instance, within the TME, specific chemokines facilitate the recruitment of immunosuppressive cell populations—including regulatory T cells (Tregs) and myeloid-derived suppressor cells (MDSCs)—which in turn promote immune evasion, tumor progression, and metastasis (23). The central role of chemokines in both health and disease underscores their significant potential as therapeutic targets for immunomodulation.

## 4 Macrophage-derived chemokines

**Figure 2:**
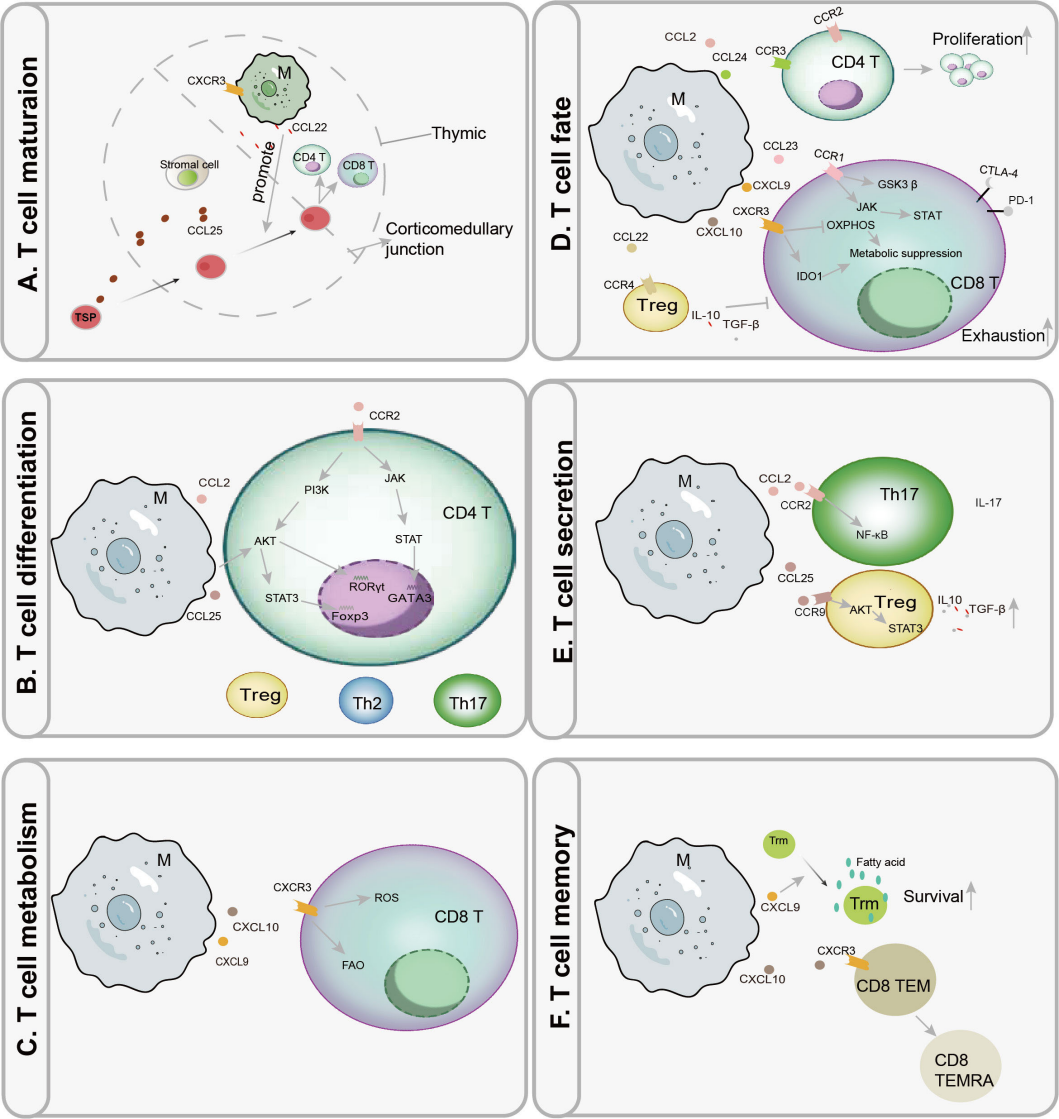
Macrophage-derived chemokines orchestrate T cell homeostasis, differentiation, and function. **(A)** Stromal cell-derived CCL25 facilitates the entry of thymus-seeding progenitors (TSPs) into the thymus. Subsequently, macrophage (M)-derived CCL22 guides their migration to the corticomedullary junction, the site for CD4^+^ and CD8^+^ T cell lineage commitment. CX3CR1^+^ macrophages contribute to central tolerance by supporting the negative selection of self-reactive T cells. **(B)** Macrophage-derived CCL2 binding to CCR2 activates the JAK-STAT/GATA3 pathway to promote Th2 polarization. Concurrently, CCL2/CCR2 signaling via the PI3K-AKT pathway upregulates RORγt to drive Th17 differentiation. In contrast, CCL25 signaling through AKT/STAT3/Foxp3^+^ facilitates regulatory T cell (Treg) development. **(C)** The CXCL9/10-CXCR3 axis enhances reactive oxygen species (ROS) levels and promotes fatty acid oxidation (FAO) in CD8^+^ T cells, optimizing them for effector functions. **(D)** Macrophage-derived CCL2/CCR2 and CCL24/CCR3 signaling promotes CD4^+^ T cell expansion. Conversely, CCL23/CCR1 engagement upregulates immune checkpoint molecules (CTLA-4, PD-1) on CD8^+^ T cells via GSK3β or JAK-STAT pathways, promoting exhaustion. Additionally, CXCL9/10 signaling *via* CXCR3 induces a metabolically suppressive state by downregulating oxidative phosphorylation (OXPHOS) and enhancing indoleamine 2,3-dioxygenase 1 (IDO1)-mediated tryptophan degradation. CCL22/CCR4 signaling stimulates Tregs to produce immunosuppressive cytokines (IL-10, TGF-β), indirectly inhibiting CD8^+^ T cell proliferation. **(E)** Macrophages activate NF-κB via CCL2/CCR2 to stimulate IL-17 production in Th17 cells. Through CCL25/CCR9-mediated AKT/STAT3 activation, they promote the secretion of IL-10 and TGF-β by Tregs. **(F)** Macrophage-secreted CXCL9 recruits tissue-resident memory T cells (Trm) to survival niches rich in fatty acids. Furthermore, CXCL10/CXCR3 signaling drives the differentiation of CD8^+^ effector memory T cells (TEM) into terminally differentiated effector cells (TEMRA).

Macrophages are integral architects of T cell homeostasis, primarily through their regulated secretion of chemokines, as depicted in **Figure 2**. This secretory activity critically shapes multiple facets of T cell biology, from development to effector function. Accordingly, this section will dissect the specific mechanisms by which macrophage-derived chemokines modulate pivotal T cell activities. We will highlight their indispensable, yet context-dependent, roles in maintaining immune equilibrium and in driving the pathogenesis of various diseases.

### 4.1 Macrophage-derived chemokines orchestrate thymocyte migration and selection

The thymus-seeding progenitors (TSPs) to the thymus are guided by stromal cell-derived CCL25, a crucial first step in T cell ontogeny that enables their differentiation into early T cell precursors (24). Following this entry, the migratory journey continues, as macrophage-derived CCL22 directs immature double-positive (CD4^+^ CD8^+^) T cells to the corticomedullary junction for subsequent pre-maturation events (2, 25). As illustrated in **Figure 2**, CX3CR1^+^ macrophages contribute to central tolerance by facilitating the negative selection of self-reactive thymocytes through antigen presentation (26). Beyond selection, these macrophages also promote T cell proliferation *via* the phagocytic clearance of apoptotic cells and bolster thymic epithelial cell (TEC)-T cell crosstalk through the secretion of CXCL16 and CCL2 (27, 28). The final stage of thymic development involves the egress of mature, naïve T cells; this process is triggered by CCL19 and CCL21, which activate Rac/RhoA signaling pathways to mediate their departure into the peripheral lymphatic system, thereby ensuring systemic distribution and establishing a pool of cells primed for immune surveillance (29–31).

### 4.2 Chemokine cues from macrophages direct CD4^+^ T cell differentiation

Following activation, naïve CD4^+^ and CD8^+^ T cells undergo differentiation into specialized effector subsets to orchestrate targeted immune responses. This critical process is potently modulated by chemokines secreted by macrophages (1, 32), as illustrated in **Figure 2**. As shown in **Figure 2**, the differentiation of CD4^+^ T cells into subsets like Treg, Th2, or Th17 is influenced by chemokines secreted by macrophages. For example, CXCL10 produced by M1-like macrophages promotes Th1 differentiation, while CCL17 from M2-like macrophages drives Th2 polarization.

As shown in **Figure 2**, macrophage-derived chemokines, like CCL2, CCL25, play a critical role in guiding the differentiation of CD4^+^ T cell subsets, including Treg, Th2, and Th17 cells. Signaling through the CCL2/CCR2 axis, for instance, can promote Th1 differentiation by upregulating the expression of key cytokines IFN-γ and IL-12 (33, 34). Furthermore, CCL2/CCR2 engagement activates downstream JAK/STAT signaling cascades, which regulate the master transcription factors T-bet and GATA3 to drive Th2 cell polarization (35, 36). Other macrophage-derived chemokines, including CXCL9, CXCL10, and CCL20, are also instrumental in stimulating effector T cell differentiation (37–39). The source of these signals is critical; notably, CXCL10 produced by pro-inflammatory M1 macrophages robustly drives Th1 polarization, thereby enhancing cell-mediated immunity (40). Conversely, M2 macrophages—often characterized by upregulated CD36 or histamine receptors—are a significant source of CCL17, a chemokine that promotes Th2 polarization and reinforces anti-inflammatory responses (41, 42).

The differentiation of Th17 cells is similarly regulated by chemokine cues. CCL2 facilitates Th17 commitment by modulating the local concentrations of TGF-β and IL-6, which in turn activate the PI3K/AKT/RORγt pathway (43, 44). The regulatory role of macrophage chemokines is complex and often indirect. Intriguingly, reduced secretion of CCL2, CCL3, CCL4, CCL5, and CXCL10 by M1 macrophages has been correlated with an expanded peripheral Treg population, underscoring the nuanced, often inhibitory role of these signals in regulatory T cell biology (45). In contrast, direct promotion of Treg development occurs *via* macrophage-derived CCL25, which activates AKT/STAT3/Foxp3 signaling to foster Treg differentiation while concurrently suppressing Fas/FasL-mediated apoptosis in contexts such as endometrial metaplasia (46). Additionally, CXCL1 supports Treg development through activation of the NF-κB/Foxp3 pathway (47).

Beyond these direct mechanisms, macrophage-derived chemokines can shape T cell fate indirectly by remodeling the local microenvironment. A key example is provided by islet macrophages, which express CXCL16 to scavenge oxidized low-density lipoproteins; this metabolic clearance activity indirectly creates a microenvironment conducive to CD8^+^ T cell differentiation (48).

### 4.3 Macrophage-derived chemokines regulate T cell immunometabolism

Cellular metabolism provides the fundamental biochemical foundation for all T cell functions, fueling processes from activation and proliferation to the execution of effector duties (49–54). The burgeoning field of immunometabolism has more recently identified chemokine signaling as a pivotal regulator of these metabolic programs (55, 56). A key mechanism involves the CXCL9/10–CXCR3 axis, which augments the cytotoxic potential of CD8^+^ T cells by elevating reactive oxygen species (ROS) and enhancing fatty acid oxidation (FAO) (57, 58). Paradoxically, this same signaling pathway can simultaneously suppress broader lipid metabolic pathways, illustrating the complex and nuanced nature of chemokine-mediated metabolic control. Beyond direct effects on T cells, macrophages can impose metabolic constraints through environmental remodeling. For instance, macrophage-derived CCL8 fosters a hypoxic niche by promoting aerobic glycolysis. This metabolic reprogramming of the TME functionally excludes cytotoxic T lymphocytes (CTLs) and suppresses their anti-tumor activity (5). Despite these advances, the mechanisms through which macrophage-secreted chemokines influence the metabolism of CD4^+^ T helper, Treg, and other lymphoid subsets represent a significant and promising frontier for future research.

### 4.4 Dual roles of macrophage-derived chemokines in T cell proliferation and exhaustion

The functional efficacy of T cells is intrinsically linked to their local abundance. In concert with survival cytokines, chemokines are critical mediators of T cell expansion and proliferation (59). For instance, macrophage-derived CCL24 acting through CCR3, or CCL2 signaling *via* CCR4, can activate the proliferation of naïve CD4^+^ T cells (60).

However, the role of macrophage-derived chemokines is not universally proliferative. In some contexts, they can suppress T cell numbers. For example, CCL18 secreted by M2 macrophages has been shown to indirectly reduce CD4^+^ T cell abundance in HIV-1 patients (61). A primary mechanism for this reduction is the induction of T cell exhaustion. As illustrated in **Figure 2**, chemokines, such as CXCL9 and CXCL10, play a crucial role in mediating T cell exhaustion, particularly in the TME, by upregulating exhaustion markers, including PD-1 on CD8^+^ T cells (62). This process is mediated through several distinct pathways. Firstly, certain chemokines directly upregulate exhaustion-associated proteins on T cells (63, 64). Macrophage-derived CCL23, CXCL9/10/11, and CCL5 upregulate CTLA-4 or PD-1 on CD8^+^ T cells *via* phosphorylation of glycogen synthase kinase 3β (GSK3β) or activation of the JAK/STAT pathway (56, 65, 66). Chemokines can alter the metabolic microenvironment to induce exhaustion (67). Overexpression of CXCL9 and CXCL10 by macrophages leads to the downregulation of OXPHOS and enhances indoleamine 2,3-dioxygenase 1 (IDO1)-mediated tryptophan degradation, thereby depleting nutrients essential for T cell proliferation (56). Furthermore, indirect mechanisms also play a key role. Stimulation through CCL22/CCR4 promotes the production of immunosuppressive factors such as IL-10 and TGF-β by Tregs; CCL8 establishes a hypoxic niche that indirectly suppresses CD8^+^ T cell expansion (5, 68).

### 4.5 Regulation of T cell cytokine secretion by macrophage-derived chemokine signaling

The secretion of effector molecules is a fundamental mechanism by which T cells execute their immune functions. A growing body of evidence indicates that macrophage-derived chemokines are potent regulators of this secretory activity. For example, signaling through CCL22 or the CXCL10/CXCR3 axis enhances IFN-γ production in T cells, a response that can exacerbate disease pathogenesis (40, 69). The underlying mechanisms involve the activation of distinct intracellular signaling pathways. In Th17 cells, CCL2 promotes IL-17 production by activating NF-κB. This IL-17 then creates a positive feedback loop by stimulating macrophages to secrete CCL20 through the NF-κB/MAPK/PI3K cascade, thereby facilitating the recruitment of more CCR6^+^ Th17 cells to the site (43, 44, 70). Conversely, a contrasting immunomodulatory effect is seen with CCL25, which activates the AKT/STAT3 pathway in Tregs, inducing the overexpression of anti-inflammatory cytokines like IL-10 and TGF-β (46). As depicted in **Figure 2**, the regulatory effects of chemokines can be influenced by macrophage polarization. M1-like macrophages typically enhance immune responses, while M2-like macrophages suppress them, contributing to immune evasion in the TME, suggesting a context-dependent mechanism of chemokine-mediated immunomodulation. Collectively, these findings demonstrate that macrophage-derived chemokines can polarize T cell responses in either a pro-inflammatory or anti-inflammatory direction. This dichotomous effect is likely determined by the phenotypic state of the macrophage source, highlighting a sophisticated, context-dependent mechanism of immunomodulation.

### 4.6 Macrophage-derived chemokines recruiting suppressive and effector T cells

T cell migration is a highly orchestrated process directed by chemokine gradients. These gradients activate GPCR signaling pathways, triggering cytoskeletal reorganization that enables targeted movement (71–75). This mechanism is fundamental not only for routine immunological surveillance but also serves as a key indicator of immune cell infiltration in pathological states (76). Macrophages regulate T cell recruitment by secreting a diverse array of chemokines that specifically attract distinct T cell subsets. For instance, CCL2 and CCL22 selectively recruit Tregs, while CXCL9 and CXCL10 are pivotal for attracting effector CD8^+^ T cells into tumors, thereby enhancing anti-tumor immunity.

Within the TME, this recruitment is often co-opted to promote immunosuppression. For example, TAM-derived CCL2 enhances the infiltration of Tregs, thereby reinforcing an immunosuppressive state (77). Similarly, macrophage-secreted CCL22 potently enhances Treg chemotaxis—a process modulated by type I interferon signaling or the nuclear translocation of p65/NF-κB (78–80). The expression level of CCL22 by M2 macrophages can predict CD4^+^ T cell abundance in the TME, and intriguingly, its expression is positively regulated by Treg-derived CXCL18, suggesting a cross-regulatory loop (81–83). Furthermore, CCL22 also facilitates the infiltration of CCR4^+^ Th2 cells in breast cancer, promoting a pro-tumorigenic immune contexture (84).

In contrast to these immunosuppressive roles, other macrophage-derived chemokines are critical for anti-tumor immunity. The CXCL9 and CXCL10 chemokines act synergistically to modulate CD8^+^ T cell chemotaxis and are essential for effective immune surveillance (85). Specifically, CXCL9 and CXCL10 derived from M1 macrophages play a critical role in promoting the migration of CD8^+^ T cells into tumors, a process indispensable for the successful killing of tumor cells (86–88). CXCL9 further cooperates with CCL5 to enhance the infiltration of CD8^+^ T cells into solid tumors (37).

CCL5 itself enhances the accumulation of CD8^+^ T cells *via* binding to its receptor, CCR5, thereby amplifying anti-tumor immune responses (89). These roles for CCL5 have been characterized not only in cancer but also in inflammatory diseases such as atherosclerosis and arthritis, and are frequently associated with the activation of inflammatory signaling pathways like NF-κB (90–93). Macrophage plasticity, exemplified by the ability of macrophages to switch between pro-inflammatory and anti-inflammatory states, introduces a dynamic regulatory mechanism that shapes the immune microenvironment. This plasticity enables macrophages to adapt their chemokine secretion patterns in response to different stimuli, influencing T cell differentiation, polarization, and functional outcomes in both tumorigenic and inflammatory contexts. Further studies indicated that repolarization from an M2-like to an M1-like phenotype could lead to a marked upregulation of CCL5 expression, resulting in the increased CD8^+^ T cell tissue infiltration (94).

### 4.7 Macrophage-derived chemokines orchestrate memory T cell differentiation and survival

The formation of memory T cells is essential for long-term adaptive immunity, enabling a rapid and potent response upon pathogen re-exposure. This process is critically dependent on chemokine-mediated recruitment (95). During the inflammatory phase, macrophages secrete chemokines that direct the migration of naïve T cells to secondary lymphoid organs, which are key sites for the initial differentiation of memory precursors (95). Additionally, monocyte-derived macrophages produce chemokines such as CCL5, CXCL9, and CXCL10, which are vital for the positioning, survival, and differentiation of tissue-resident memory T cell (Trm) precursors (96, 97).

M1 macrophages contribute to this process by producing CXCL9, which recruits CD8^+^ Trm cells to inflammatory sites enriched with fatty acids that support T cell survival (98). A fascinating positive feedback loop exists within the TME: CD8^+^ Trm cells promote CXCL9 transcription in macrophages *via* the IFN-γ/p-STAT1 axis, which in turn reinforces Trm retention and survival (99). This mechanism ensures swift responses to recurrent infections and enhances durable immune surveillance.

The therapeutic relevance of these pathways is increasingly apparent. Collectively, this chemokine-driven regulation of memory T cell recruitment, differentiation, and survival underscores the central role of macrophages in bridging innate and adaptive immunity, ensuring a potent and rapid response to recurrent challenges.

## 5 Cancer

Within the TME, the anti-tumor efficacy of CD8^+^ T lymphocytes is frequently compromised by both a decline in their absolute numbers and a state of functional impairment. This dysfunction inhibits their primary killing mechanisms, which include the perforin-granzyme/caspase cascade and IFN-γ-mediated pathways (100, 101). This immunosuppressed state is further exacerbated by Tregs, which secrete inhibitory molecules like IL-10 and TGF-β and upregulate checkpoint ligands that suppress the perforin-granzyme axis (100, 102, 103).

The polarization state of tumor-infiltrating macrophages is a critical determinant of this balance. In recent years, the divergent roles of pro-inflammatory M1 macrophages and immunosuppressive TAMs have garnered significant attention. Polarization towards the TAM phenotype, driven by tumor-derived signals and metabolic abnormalities, directly and indirectly exacerbates immunosuppression. In stark contrast, M1 macrophages typically counteract these effects and promote immune activation (104, 105). This section will focus on the mechanisms by which these two macrophage subsets differentially modulate the tumor-killing capacity of CD8^+^ T cells through their secretion of chemokines.

### 5.1 TAM-derived chemokines facilitate CD8^+^ T cell dysfunction

**Figure 3:**
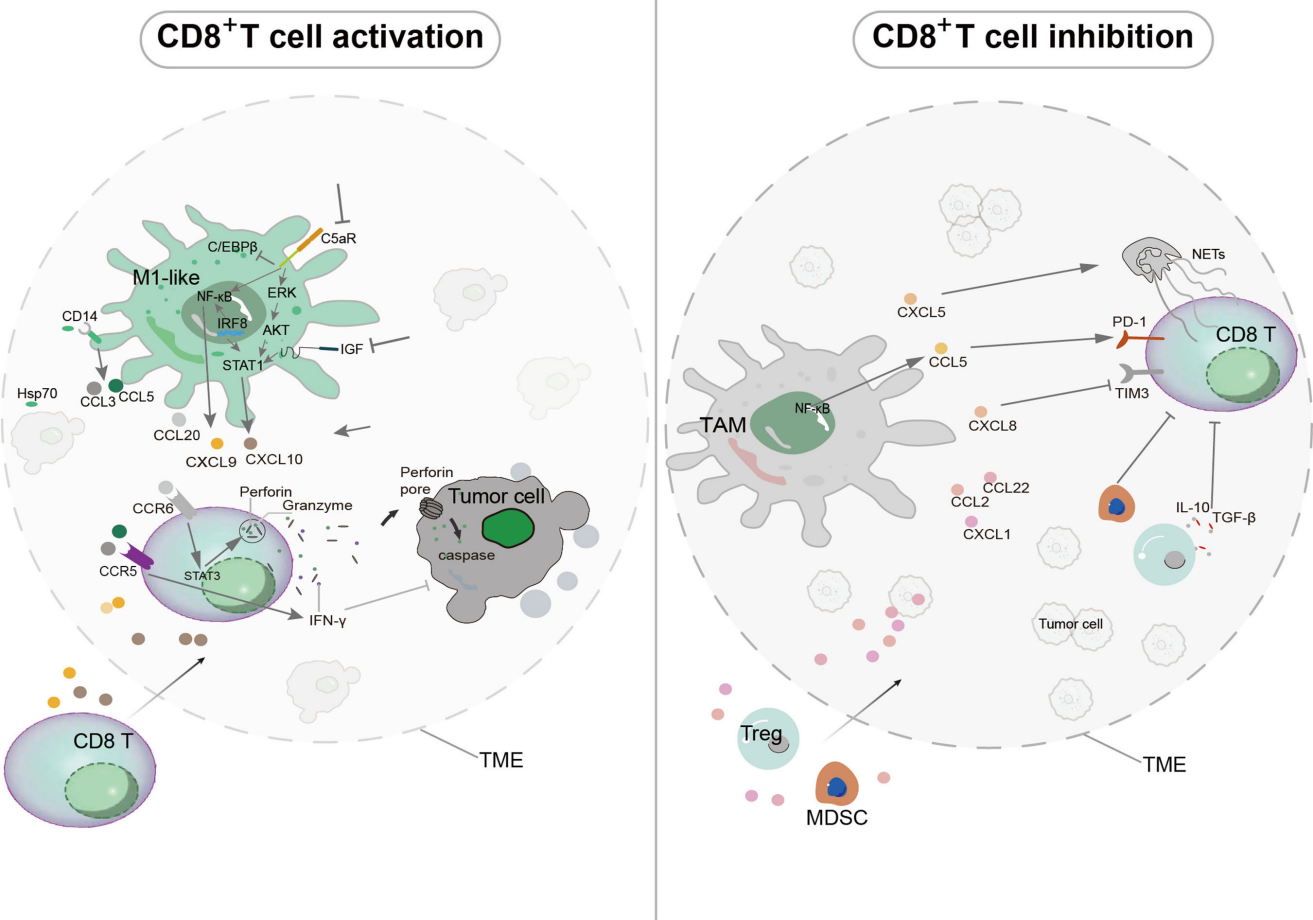
Tumor-associated macrophages (TAMs) and M1 macrophages regulate CD8^+^ T cell-mediated tumor immunity through chemokine signaling. (CD8^+^ T cell activation). Pharmacological inhibition of C5aR enhances phosphorylation of ERK, AKT, and NF-κB, while suppressing C/EBPβ phosphorylation. Blockade of insulin-like growth factor (IGF) signaling upregulates STAT1, and overexpression of IRF8 enhances both NF-κB and STAT1 signaling. These interventions promote the secretion of CXCL9 and CXCL10 by M1 macrophages, which facilitate CD8^+^ T cell recruitment into the tumor microenvironment (TME). M1 macrophage-derived CCL20 binds to CCR6 and activates STAT3, stimulating CD8^+^ T cells to produce perforin. Granzyme then enters tumor cells through perforin pores, initiating caspase-mediated apoptosis. Tumor-derived Hsp70 (heat shock protein 70) triggers CCL3 and CCL5 secretion from M1-like macrophages *via* CD14 activation, and the released CCL3 and CCL5 stimulate IFN-γ production in CD8^+^ T cells through CCR5 signaling. (CD8^+^ T cell inhibition) TAM-derived CCL2, CCL22, and CXCL1 recruit myeloid-derived suppressor cells (MDSCs) or regulatory T cells (Tregs) — which secrete IL-10 and TGF-β — into the TME (dotted circle), thereby inhibiting CD8^+^ T cell function. CXCL5 stimulates neutrophil extracellular trap (NET) formation, leading to CD8^+^ T cell exhaustion. CCL5 upregulates the exhaustion marker PD-1 on CD8^+^ T cells, while CXCL8 downregulates the survival marker TIM-3.

TAMs employ a repertoire of chemokines to enact immunosuppressive programs that ultimately inhibit CD8^+^ T cell function (**Figure 3**). A primary strategy involves the indirect suppression of CD8^+^ T cells *via* the recruitment of other inhibitory cells. Specifically, TAM-secreted CCL2, CCL22, and CXCL1 facilitate the infiltration of Tregs and MDSCs (23, 82, 106–108). The critical role of CCL22 is underscored by studies showing that anti-CCL22 antibodies significantly reduce intratumoral Treg accumulation (109). Beyond recruitment, TAMs can drive CTL exhaustion through neutrophil engagement. For instance, via NF-κB-mediated upregulation of CXCL5, TAMs stimulate neutrophil extracellular trap (NET) formation, which ultimately promotes a state of CD8^+^ T cell exhaustion (105).

Moreover, TAM-derived chemokines can directly impair CD8^+^ T cell function and promote tumor progression. In glioblastoma, a synergistic effect between TAM-secreted CCL8 and an IL-1β-induced hypoxic microenvironment collectively suppresses CD8^+^ T cell activity (5). In clear cell renal cell carcinoma, TAM-derived CCL5 directly upregulates the expression of the exhaustion marker PD-1 on CD8^+^ T cells (110). Furthermore, in advanced colorectal cancer, TAMs suppress TIM-3—a critical molecule for CD8^+^ T cell proliferation and effector function—through the IL-8 (CXCL8)/CXCR2 axis (111). These findings collectively underscore the pivotal role of TAM-derived chemokines in facilitating tumor immune escape.

### 5.2 M1 macrophage-derived chemokines enhance the tumor-killing capacity of CD8^+^ T cells

In direct opposition to TAMs, M1 macrophages secrete chemokines that are critical for initiating and sustaining effective anti-tumor T cell responses, making them attractive therapeutic targets (**Figure 3**). A key function of M1 macrophages is the production of CXCL9, CXCL10, and CXCL11, which facilitate the recruitment and intratumoral positioning of CXCR3-expressing CD8^+^ T cells. This process is fundamental for establishing a favorable microenvironment for immune checkpoint inhibitor therapies, such as anti-PD-(L)1 treatment (112, 113). In the context of atezolizumab plus bevacizumab combination therapy for advanced hepatocellular carcinoma, macrophage-derived CXCL10 promotes the preferential differentiation of CXCR3^+^ CD8^+^ effector memory T cells into highly cytotoxic PD1^−^ CD45RA^+^ terminal effector cells (TEMRA) (88).

The signaling pathways that drive the production of these chemokines are diverse and can be therapeutically modulated. For example, in ovarian cancer, pharmacological inhibition of the immunosuppressive C5a receptor enhances phosphorylation of ERK, AKT, and NF-κB while suppressing C/EBPβ phosphorylation, resulting in increased CXCL9 production (114). Similarly, blockade of insulin-like growth factor (IGF) signaling upregulates STAT1 transcription and inhibits STAT3 phosphorylation in M1 macrophages, thereby promoting the expression of CXCL9 and CXCL10 (115). In colorectal cancer liver metastases, microwave ablation combined with αPD-L1 therapy upregulates the transcription factor IRF8, which enhances both NF-κB and JAK-STAT1 signaling to stimulate CXCL9 production in M1 macrophages (116).

Beyond recruitment, M1 macrophage-derived chemokines can directly augment the cytotoxic arsenal of CD8^+^ T cells. Macrophage-secreted CCL20 binds to CCR6 (CD196) on CD8^+^ T cells, promoting STAT3-dependent transcription of perforin and enhancing its expression (117). Tumor-derived Hsp70 (heat shock protein 70) triggers CCL3 and CCL5 secretion from M1-like macrophages *via* CD14 activation, and the released CCL3 and CCL5 stimulate IFN-γ production in CD8^+^ T cells through CCR5 signaling (118).

This anti-tumor activity is reinforced by powerful cytokine feedback loops. Elevated levels of IFN-γ within the TME, often secreted by activated T cells, stimulate M1 macrophages to further increase their secretion of CXCL9 and CXCL10. This creates a positive feedback loop that amplifies T cell recruitment and enhances tumor clearance (119). The expression profile of these macrophage-derived chemokines has even been proposed as an independent prognostic biomarker in cancer, offering valuable insights for designing personalized treatment strategies (110, 120). Yi et al. (121) summarized the current understanding and therapeutic implications of targeting cytokine and chemokine signaling pathways in cancer. By exploring the roles of these molecules in tumor biology and the immune response, they highlighted the development of novel therapeutic agents aimed at modulating these pathways to combat cancer.

## 6 Conclusions

This review has elucidated the complex mechanisms through which macrophage-derived chemokines govern T cell biology, emphasizing their context-dependent roles in the TME. It highlighted how chemokines from immunosuppressive TAMs, such as CCL2 and CCL22, could mediate immune evasion by recruiting regulatory cell populations and promoting T cell exhaustion. In contrast, chemokines, such as CXCL9 and CXCL10 secreted by pro-inflammatory macrophages are pivotal in driving anti-tumor immunity by enhancing the infiltration and activation of cytotoxic CD8^+^ T cells. Consequently, targeting these chemokine-mediated regulatory mechanisms represents a notable direction for the development of novel cancer immunotherapies.

Although the traditional M1/M2 macrophage paradigm has provided useful insights, recent advances in single-cell transcriptomics have revealed a more complex and diverse spectrum of macrophage phenotypes. Previous research has demonstrated that macrophage populations exhibit significant heterogeneity, and their functional roles in immunity and tumor progression cannot be fully captured by the M1/M2 model. Therefore, understanding this macrophage diversity is crucial for refining therapeutic strategies.

Moreover, indirect mechanisms, including metabolic reprogramming pathways, such as IDO1-mediated tryptophan degradation, remain underexplored, while are critical in modulating T cell responses in the TME. To address these challenges, future research must utilize cutting-edge tools, including single-cell transcriptomics, spatial transcriptomics, and multiplexed imaging. These technologies, which have already provided valuable insights into macrophage diversity, are essential for deconvoluting specific macrophage subsets, mapping their interactions with T cells, and profiling their chemokine expression patterns in spatial contexts. Targeting these chemokine-mediated regulatory mechanisms represents a remarkably promising direction for the development of novel cancer immunotherapies. This comprehensive approach may pave the way for more precise and targeted immunotherapies that exploit chemokine networks, overcoming current limitations and employing the full potential of the immune system to combat cancer.

## References

[1] Mantovani A Sica A Sozzani S Allavena P Vecchi A Locati M . The chemokine system in diverse forms of macrophage activation and polarization. Trends Immunol. (2004) 12:677–86. doi: 10.1016/j.it.2004.09.015, PMID: 15530839

[2] McCabe A MacNamara KC . Macrophages: Key regulators of steady-state and demand-adapted hematopoiesis. Exp Hematol. (2016) 4:213–22. doi: 10.1016/j.exphem.2016.01.003, PMID: 26806720 PMC4852701

[3] Norment AM Bevan MJ . Role of chemokines in thymocyte development. Semin Immunol. (2000) 5:445–55. doi: 10.1006/smim.2000.0261, PMID: 11085177

[4] Van Raemdonck K Umar S Palasiewicz K Volkov S Volin MV Arami S . CCL21/CCR7 signaling in macrophages promotes joint inflammation and Th17-mediated osteoclast formation in rheumatoid arthritis. Cell Mol Life Sci. (2020) 7:1387–99. doi: 10.1007/s00018-019-03235-w, PMID: 31342120 PMC10040247

[5] Sattiraju A Kang S Giotti B Chen Z Marallano VJ Brusco C . Hypoxic niches attract and sequester tumor-associated macrophages and cytotoxic T cells and reprogram them for immunosuppression. Immunity. (2023) 8:1825–43.e6. doi: 10.1016/j.immuni.2023.06.017, PMID: 37451265 PMC10527169

[6] Liu N Wang X Steer CJ Song G . MicroRNA-206 promotes the recruitment of CD8(+) T cells by driving M1 polarisation of Kupffer cells. Gut. (2022) 8:1642–55. doi: 10.1136/gutjnl-2021-324170, PMID: 34706869 PMC9279850

[7] Epelman S Lavine KJ Randolph GJ . Origin and functions of tissue macrophages. Immunity. (2014) 1:21–35. doi: 10.1016/j.immuni.2014.06.013, PMID: 25035951 PMC4470379

[8] Locati M Curtale G Mantovani A . Diversity, mechanisms, and significance of macrophage plasticity. Annu Rev Pathol. (2020) 15):123–47. doi: 10.1146/annurev-pathmechdis-012418-012718, PMID: 31530089 PMC7176483

[9] Luo M Zhao F Cheng H Su M Wang Y . Macrophage polarization: an important role in inflammatory diseases. Front Immunol. (2024) 15):1352946. doi: 10.3389/fimmu.2024.1352946, PMID: 38660308 PMC11039887

[10] Xia T Zhang M Lei W Yang R Fu S Fan Z . Advances in the role of STAT3 in macrophage polarization. Front Immunol. (2023) 14):1160719. doi: 10.3389/fimmu.2023.1160719, PMID: 37081874 PMC10110879

[11] Jain N Moeller J Vogel V . Mechanobiology of macrophages: how physical factors coregulate macrophage plasticity and phagocytosis. Annu Rev BioMed Eng. (2019) 21):267–97. doi: 10.1146/annurev-bioeng-062117-121224, PMID: 31167103

[12] Sun L Su Y Jiao A Wang X Zhang B . T cells in health and disease. Signal Transduct Target Ther. (2023) 1:235. doi: 10.1038/s41392-023-01471-y, PMID: 37332039 PMC10277291

[13] Kadomoto S Izumi K Mizokami A . Macrophage polarity and disease control. Int J Mol Sci. (2021) 1:144. doi: 10.3390/ijms23010144, PMID: 35008577 PMC8745226

[14] Guerriero JL . Macrophages: their untold story in T cell activation and function. Int Rev Cell Mol Biol. (2019) 342):73–93. doi: 10.1016/bs.ircmb.2018.07.001, PMID: 30635094

[15] van der Vorst EP Döring Y Weber C . Chemokines. Arterioscler Thromb Vasc Biol. (2015) 11:e52–6. doi: 10.1161/atvbaha.115.306359, PMID: 26490276

[16] Fernandez EJ Lolis E . Structure, function, and inhibition of chemokines. Annu Rev Pharmacol Toxicol. (2002) 42):469–99. doi: 10.1146/annurev.pharmtox.42.091901.115838, PMID: 11807180

[17] Hauser MA Legler DF . Common and biased signaling pathways of the chemokine receptor CCR7 elicited by its ligands CCL19 and CCL21 in leukocytes. J Leukoc Biol. (2016) 6:869–82. doi: 10.1189/jlb.2MR0815-380R, PMID: 26729814

[18] Hughes CE Nibbs RJB . A guide to chemokines and their receptors. FEBS J. (2018) 16:2944–71. doi: 10.1111/febs.14466, PMID: 29637711 PMC6120486

[19] Nagai M Noguchi R Takahashi D Morikawa T Koshida K Komiyama S . Fasting-refeeding impacts immune cell dynamics and mucosal immune responses. Cell. (2019) 5:1072–87.e14. doi: 10.1016/j.cell.2019.07.047, PMID: 31442401

[20] Rahimi RA Luster AD . Chemokines: critical regulators of memory T cell development, maintenance, and function. Adv Immunol. (2018) 138):71–98. doi: 10.1016/bs.ai.2018.02.002, PMID: 29731007 PMC6191293

[21] Correia AL Guimaraes JC Auf der Maur P De Silva D Trefny MP Okamoto R . Hepatic stellate cells suppress NK cell-sustained breast cancer dormancy. Nature. (2021) 7864:566–71. doi: 10.1038/s41586-021-03614-z, PMID: 34079127

[22] Vilgelm AE Richmond A . Chemokines modulate immune surveillance in tumorigenesis, metastasis, and response to immunotherapy. Front Immunol. (2019) 10):333. doi: 10.3389/fimmu.2019.00333, PMID: 30873179 PMC6400988

[23] Ozga AJ Chow MT Luster AD . Chemokines and the immune response to cancer. Immunity. (2021) 5:859–74. doi: 10.1016/j.immuni.2021.01.012, PMID: 33838745 PMC8434759

[24] Jenkinson WE Rossi SW Parnell SM Agace WW Takahama Y Jenkinson EJ . Chemokine receptor expression defines heterogeneity in the earliest thymic migrants. Eur J Immunol. (2007) 8:2090–6. doi: 10.1002/eji.200737212, PMID: 17578846

[25] Zhang Y Zhang C Feng R Meng T Peng W Song J . CXCR4 regulates macrophage M1 polarization by altering glycolysis to promote prostate fibrosis. Cell Commun Signal. (2024) 1:456. doi: 10.1186/s12964-024-01828-y, PMID: 39327570 PMC11426013

[26] Wang H Zúñiga-Pflücker JC . Thymic microenvironment: interactions between innate immune cells and developing thymocytes. Front Immunol. (2022) 13):885280. doi: 10.3389/fimmu.2022.885280, PMID: 35464404 PMC9024034

[27] Zhou TA Hsu HP Tu YH Cheng HK Lin CY Chen NJ . Thymic macrophages consist of two populations with distinct localization and origin. Elife. (2022) 11:e75148. doi: 10.7554/eLife.75148, PMID: 36449334 PMC9754631

[28] Baratin M Simon L Jorquera A Ghigo C Dembele D Nowak J . T cell zone resident macrophages silently dispose of apoptotic cells in the lymph node. Immunity. (2017) 2:349–62.e5. doi: 10.1016/j.immuni.2017.07.019, PMID: 28801233

[29] Ruiz Pérez M Vandenabeele P Tougaard P . The thymus road to a T cell: migration, selection, and atrophy. Front Immunol. (2024) 15):1443910. doi: 10.3389/fimmu.2024.1443910, PMID: 39257583 PMC11384998

[30] Mou F Praskova M Xia F Van Buren D Hock H Avruch J . The Mst1 and Mst2 kinases control activation of rho family GTPases and thymic egress of mature thymocytes. J Exp Med. (2012) 4:741–59. doi: 10.1084/jem.20111692, PMID: 22412158 PMC3328371

[31] Ueno T Hara K Willis MS Malin MA Höpken UE Gray DH . Role for CCR7 ligands in the emigration of newly generated T lymphocytes from the neonatal thymus. Immunity. (2002) 2:205–18. doi: 10.1016/s1074-7613(02)00267-4, PMID: 11869682

[32] Yao Y Xue H Chen X Cao Y Yu J Jiang X . Polarization of helper T lymphocytes maybe involved in the pathogenesis of lumbar disc herniation. Iran J Allergy Asthma Immunol. (2017) 4:347–57. Available online at: https://pubmed.ncbi.nlm.nih.gov/28865415/, PMID: 28865415

[33] Xu M Wang Y Xia R Wei Y Wei X . Role of the CCL2-CCR2 signalling axis in cancer: Mechanisms and therapeutic targeting. Cell Prolif. (2021) 10:e13115. doi: 10.1111/cpr.13115, PMID: 34464477 PMC8488570

[34] Fei L Ren X Yu H Zhan Y . Targeting the CCL2/CCR2 axis in cancer immunotherapy: one stone, three birds? Front Immunol. (2021) 12):771210. doi: 10.3389/fimmu.2021.771210, PMID: 34804061 PMC8596464

[35] Hao Q Vadgama JV Wang P . CCL2/CCR2 signaling in cancer pathogenesis. Cell Commun Signal. (2020) 1:82. doi: 10.1186/s12964-020-00589-8, PMID: 32471499 PMC7257158

[36] Lin Z Shi JL Chen M Zheng ZM Li MQ Shao J . CCL2: An important cytokine in normal and pathological pregnancies: A review. Front Immunol. (2022) 13):1053457. doi: 10.3389/fimmu.2022.1053457, PMID: 36685497 PMC9852914

[37] Dangaj D Bruand M Grimm AJ Ronet C Barras D Duttagupta PA . Cooperation between constitutive and inducible chemokines enables T cell engraftment and immune attack in solid tumors. Cancer Cell. (2019) 6:885–900.e10. doi: 10.1016/j.ccell.2019.05.004, PMID: 31185212 PMC6961655

[38] Kadomoto S Izumi K Mizokami A . The CCL20-CCR6 axis in cancer progression. Int J Mol Sci. (2020) 15:5186. doi: 10.3390/ijms21155186, PMID: 32707869 PMC7432448

[39] Kasraie S Niebuhr M Kopfnagel V Dittrich-Breiholz O Kracht M Werfel T . Macrophages from patients with atopic dermatitis show a reduced CXCL10 expression in response to staphylococcal α-toxin. Allergy. (2012) 1:41–9. doi: 10.1111/j.1398-9995.2011.02710.x, PMID: 21906079

[40] Liu P Jia S Lou Y He K Xu LX . Cryo-thermal therapy inducing MI macrophage polarization created CXCL10 and IL-6-rich pro-inflammatory environment for CD4(+) T cell-mediated anti-tumor immunity. Int J Hyperthermia. (2019) 1:408–20. doi: 10.1080/02656736.2019.1579373, PMID: 30892102

[41] Zhou ZR Fang SB Liu XQ Li CG Xie YC He BX . Serum amyloid A1 induced dysfunction of airway macrophages via CD36 pathway in allergic airway inflammation. Int Immunopharmacol. (2024), 142(Pt A):113081. doi: 10.1016/j.intimp.2024.113081, PMID: 39244902

[42] Mommert S Gregor K Rossbach K Schaper K Witte T Gutzmer R . Histamine H2 receptor stimulation upregulates T(H)2 chemokine CCL17 production in human M2a macrophages. J Allergy Clin Immunol. (2018) 2:782–5.e5. doi: 10.1016/j.jaci.2017.06.023, PMID: 28728999

[43] Bakos E Thaiss CA Kramer MP Cohen S Radomir L Orr I . CCR2 regulates the immune response by modulating the interconversion and function of effector and regulatory T cells. J Immunol. (2017) 12:4659–71. doi: 10.4049/jimmunol.1601458, PMID: 28507030

[44] Kaushansky N Bakos E Becker-Herman S Shachar I Ben-Nun A . Circulating picomolar levels of CCL2 downregulate ongoing chronic experimental autoimmune encephalomyelitis by induction of regulatory mechanisms. J Immunol. (2019) 7:1857–66. doi: 10.4049/jimmunol.1900424, PMID: 31484731

[45] Mancuso RI Azambuja JH Olalla Saad ST . Artesunate strongly modulates myeloid and regulatory T cells to prevent LPS-induced systemic inflammation. BioMed Pharmacother. (2021) 143):112211. doi: 10.1016/j.biopha.2021.112211, PMID: 34649344

[46] Li MQ Wang Y Chang KK Meng YH Liu LB Mei J . CD4+Foxp3+ regulatory T cell differentiation mediated by endometrial stromal cell-derived TECK promotes the growth and invasion of endometriotic lesions. Cell Death Dis. (2014) 10:e1436. doi: 10.1038/cddis.2014.414, PMID: 25275597 PMC4649519

[47] Li J Wang S Wang N Zheng Y Yang B Wang X . Aiduqing formula inhibits breast cancer metastasis by suppressing TAM/CXCL1-induced Treg differentiation and infiltration. Cell Commun Signal. (2021) 1:89. doi: 10.1186/s12964-021-00775-2, PMID: 34461944 PMC8404313

[48] Srivastava N Hu H Peterson OJ Vomund AN Stremska M Zaman M . CXCL16-dependent scavenging of oxidized lipids by islet macrophages promotes differentiation of pathogenic CD8(+) T cells in diabetic autoimmunity. Immunity. (2024) 7:1629–47.e8. doi: 10.1016/j.immuni.2024.04.017, PMID: 38754432 PMC11236520

[49] Kishton RJ Sukumar M Restifo NP . Metabolic regulation of T cell longevity and function in tumor immunotherapy. Cell Metab. (2017) 1:94–109. doi: 10.1016/j.cmet.2017.06.016, PMID: 28683298 PMC5543711

[50] Shyer JA Flavell RA Bailis W . Metabolic signaling in T cells. Cell Res. (2020) 8:649–59. doi: 10.1038/s41422-020-0379-5, PMID: 32709897 PMC7395146

[51] Almeida L Lochner M Berod L Sparwasser T . Metabolic pathways in T cell activation and lineage differentiation. Semin Immunol. (2016) 5:514–24. doi: 10.1016/j.smim.2016.10.009, PMID: 27825556

[52] Ma S Ming Y Wu J Cui G . Cellular metabolism regulates the differentiation and function of T-cell subsets. Cell Mol Immunol. (2024) 5:419–35. doi: 10.1038/s41423-024-01148-8, PMID: 38565887 PMC11061161

[53] Zhu Y Zhang X Yang K Shao Y Gu R Liu X . Macrophage-derived apoptotic vesicles regulate fate commitment of mesenchymal stem cells via miR155. Stem Cell Res Ther. (2022) 1:323. doi: 10.1186/s13287-022-03004-w, PMID: 35842708 PMC9288680

[54] O’Sullivan D . The metabolic spectrum of memory T cells. Immunol Cell Biol. (2019) 7:636–46. doi: 10.1111/imcb.12274, PMID: 31127964

[55] Sun J Zhang Y Yang M Zhang Y Xie Q Li Z . Hypoxia induces T-cell apoptosis by inhibiting chemokine C receptor 7 expression: the role of adenosine receptor A(2). Cell Mol Immunol. (2010) 1:77–82. doi: 10.1038/cmi.2009.105, PMID: 20029460 PMC4003256

[56] Pichler R Siska PJ Tymoszuk P Martowicz A Untergasser G Mayr R . A chemokine network of T cell exhaustion and metabolic reprogramming in renal cell carcinoma. Front Immunol. (2023) 14):1095195. doi: 10.3389/fimmu.2023.1095195, PMID: 37006314 PMC10060976

[57] Wan H Xu B Zhu N Ren B . PGC-1α activator-induced fatty acid oxidation in tumor-infiltrating CTLs enhances effects of PD-1 blockade therapy in lung cancer. Tumori. (2020) 1:55–63. doi: 10.1177/0300891619868287, PMID: 31451071

[58] Lieber S Reinartz S Raifer H Finkernagel F Dreyer T Bronger H . Prognosis of ovarian cancer is associated with effector memory CD8(+) T cell accumulation in ascites, CXCL9 levels and activation-triggered signal transduction in T cells. Oncoimmunology. (2018) 5:e1424672. doi: 10.1080/2162402x.2018.1424672, PMID: 29721385 PMC5927536

[59] Di Pilato M Kfuri-Rubens R Pruessmann JN Ozga AJ Messemaker M Cadilha BL . CXCR6 positions cytotoxic T cells to receive critical survival signals in the tumor microenvironment. Cell. (2021) 17:4512–30.e22. doi: 10.1016/j.cell.2021.07.015, PMID: 34343496 PMC8719451

[60] Ancuta P Autissier P Wurcel A Zaman T Stone D Gabuzda D . CD16+ monocyte-derived macrophages activate resting T cells for HIV infection by producing CCR3 and CCR4 ligands. J Immunol. (2006) 10:5760–71. doi: 10.4049/jimmunol.176.10.5760, PMID: 16670281

[61] Malhotra P Haslett P Sherry B Shepp DH Barber P Abshier J . Increased Plasma Levels of the TH2 chemokine CCL18 associated with low CD4+ T cell counts in HIV-1-infected Patients with a Suppressed Viral Load. Sci Rep. (2019) 1:5963. doi: 10.1038/s41598-019-41588-1, PMID: 30979916 PMC6461658

[62] Yang M Lin C Wang Y Chen K Han Y Zhang H . Cytokine storm promoting T cell exhaustion in severe COVID-19 revealed by single cell sequencing data analysis. Precis Clin Med. (2022) 2:pbac014. doi: 10.1093/pcmedi/pbac014, PMID: 35694714 PMC9172646

[63] Kfoury Y Baryawno N Severe N Mei S Gustafsson K Hirz T . Human prostate cancer bone metastases have an actionable immunosuppressive microenvironment. Cancer Cell. (2021) 11:1464–78.e8. doi: 10.1016/j.ccell.2021.09.005, PMID: 34719426 PMC8578470

[64] Curiel TJ Coukos G Zou L Alvarez X Cheng P Mottram P . Specific recruitment of regulatory T cells in ovarian carcinoma fosters immune privilege and predicts reduced survival. Nat Med. (2004) 9:942–9. doi: 10.1038/nm1093, PMID: 15322536

[65] Kamat K Krishnan V Dorigo O . Macrophage-derived CCL23 upregulates expression of T-cell exhaustion markers in ovarian cancer. Br J Cancer. (2022) 6:1026–33. doi: 10.1038/s41416-022-01887-3, PMID: 35750747 PMC9470573

[66] Horie M Takagane K Itoh G Kuriyama S Yanagihara K Yashiro M . Exosomes secreted by ST3GAL5(high) cancer cells promote peritoneal dissemination by establishing a premetastatic microenvironment. Mol Oncol. (2024) 1:21–43. doi: 10.1002/1878-0261.13524, PMID: 37716915 PMC10766203

[67] Franciszkiewicz K Boissonnas A Boutet M Combadière C Mami-Chouaib F . Role of chemokines and chemokine receptors in shaping the effector phase of the antitumor immune response. Cancer Res. (2012) 24:6325–32. doi: 10.1158/0008-5472.Can-12-2027, PMID: 23222302

[68] Xu X Bian L Shen M Li X Zhu J Chen S . Multipeptide-coupled nanoparticles induce tolerance in ‘humanised’ HLA-transgenic mice and inhibit diabetogenic CD8(+) T cell responses in type 1 diabetes. Diabetologia. (2017) 12:2418–31. doi: 10.1007/s00125-017-4419-8, PMID: 28887632

[69] Ushio A Arakaki R Otsuka K Yamada A Tsunematsu T Kudo Y . CCL22-producing resident macrophages enhance T cell response in sjögren’s syndrome. Front Immunol. (2018) 9):2594. doi: 10.3389/fimmu.2018.02594, PMID: 30467506 PMC6236111

[70] Zhang C Xu S Hu RJ Liu XH Yue SY Li XL . Unraveling CCL20’s role by regulating Th17 cell chemotaxis in experimental autoimmune prostatitis. J Cell Mol Med. (2024) 10:e18445. doi: 10.1111/jcmm.18445, PMID: 38801403 PMC11129727

[71] Kohli K Pillarisetty VG Kim TS . Key chemokines direct migration of immune cells in solid tumors. Cancer Gene Ther. (2022) 1:10–21. doi: 10.1038/s41417-021-00303-x, PMID: 33603130 PMC8761573

[72] Savino W Mendes-da-Cruz DA Silva JS Dardenne M Cotta-de-Almeida V . Intrathymic T-cell migration: a combinatorial interplay of extracellular matrix and chemokines? Trends Immunol. (2002) 6:305–13. doi: 10.1016/s1471-4906(02)02224-x, PMID: 12072370

[73] Ward SG . T lymphocytes on the move: chemokines, PI 3-kinase and beyond. Trends Immunol. (2006) 2:80–7. doi: 10.1016/j.it.2005.12.004, PMID: 16413226

[74] Ridley AJL Ou Y Karlsson R Pun N Birchenough HL Mulholland IZ . Chemokines form complex signals during inflammation and disease that can be decoded by extracellular matrix proteoglycans. Sci Signal. (2023) 810:eadf2537. doi: 10.1126/scisignal.adf2537, PMID: 37934811 PMC7617913

[75] Gurusamy M Tischner D Shao J Klatt S Zukunft S Bonnavion R . G-protein-coupled receptor P2Y10 facilitates chemokine-induced CD4 T cell migration through autocrine/paracrine mediators. Nat Commun. (2021) 1:6798. doi: 10.1038/s41467-021-26882-9, PMID: 34815397 PMC8611058

[76] Tang Y Gu Z Fu Y Wang J . CXCR3 from chemokine receptor family correlates with immune infiltration and predicts poor survival in osteosarcoma. Biosci Rep. (2019) 11:BSR20192134. doi: 10.1042/bsr20192134, PMID: 31696204 PMC6851512

[77] Chiang Y Lu LF Tsai CL Tsai YC Wang CC Hsueh FJ . C-C chemokine receptor 4 (CCR4)-positive regulatory T cells interact with tumor-associated macrophages to facilitate metastatic potential after radiation. Eur J Cancer. (2024) 198):113521. doi: 10.1016/j.ejca.2023.113521, PMID: 38171115

[78] Liu N Chang CW Steer CJ Wang XW Song G . MicroRNA-15a/16–1 prevents hepatocellular carcinoma by disrupting the communication between kupffer cells and regulatory T cells. Gastroenterology. (2022) 2:575–89. doi: 10.1053/j.gastro.2021.10.015, PMID: 34678217

[79] Anz D Rapp M Eiber S Koelzer VH Thaler R Haubner S . Suppression of intratumoral CCL22 by type i interferon inhibits migration of regulatory T cells and blocks cancer progression. Cancer Res. (2015) 21:4483–93. doi: 10.1158/0008-5472.Can-14-3499, PMID: 26432403

[80] Sun J Sun J Song B Zhang L Shao Q Liu Y . Fucoidan inhibits CCL22 production through NF-κB pathway in M2 macrophages: a potential therapeutic strategy for cancer. Sci Rep. (2016) 6):35855. doi: 10.1038/srep35855, PMID: 27775051 PMC5075786

[81] Zhou M Bracci PM McCoy LS Hsuang G Wiemels JL Rice T . Serum macrophage-derived chemokine/CCL22 levels are associated with glioma risk, CD4 T cell lymphopenia and survival time. Int J Cancer. (2015) 4:826–36. doi: 10.1002/ijc.29441, PMID: 25604093 PMC4478165

[82] Kimura S Nanbu U Noguchi H Harada Y Kumamoto K Sasaguri Y . Macrophage CCL22 expression in the tumor microenvironment and implications for survival in patients with squamous cell carcinoma of the tongue. J Oral Pathol Med. (2019) 8:677–85. doi: 10.1111/jop.12885, PMID: 31134686

[83] Wang D Yang L Yue D Cao L Li L Wang D . Macrophage-derived CCL22 promotes an immunosuppressive tumor microenvironment via IL-8 in Malignant pleural effusion. Cancer Lett. (2019) 452):244–53. doi: 10.1016/j.canlet.2019.03.040, PMID: 30928379

[84] Mandal PK Biswas S Mandal G Purohit S Gupta A Majumdar Giri A . CCL2 conditionally determines CCL22-dependent Th2-accumulation during TGF-β-induced breast cancer progression. Immunobiology. (2018) 2:151–61. doi: 10.1016/j.imbio.2017.10.031, PMID: 29107385

[85] Peranzoni E Lemoine J Vimeux L Feuillet V Barrin S Kantari-Mimoun C . Macrophages impede CD8 T cells from reaching tumor cells and limit the efficacy of anti-PD-1 treatment. Proc Natl Acad Sci U.S.A. (2018) 17:E4041–e50. doi: 10.1073/pnas.1720948115, PMID: 29632196 PMC5924916

[86] House IG Savas P Lai J Chen AXY Oliver AJ Teo ZL . Macrophage-derived CXCL9 and CXCL10 are required for antitumor immune responses following immune checkpoint blockade. Clin Cancer Res. (2020) 2:487–504. doi: 10.1158/1078-0432.Ccr-19-1868, PMID: 31636098

[87] Chang Z Zhang Q Hu Q Liu Y Zhang L Liu R . Tannins in Terminalia bellirica inhibits hepatocellular carcinoma growth via re-educating tumor-associated macrophages and restoring CD8(+)T cell function. BioMed Pharmacother. (2022) 154):113543. doi: 10.1016/j.biopha.2022.113543, PMID: 36057223

[88] Cappuyns S Philips G Vandecaveye V Boeckx B Schepers R Van Brussel T . PD-1(-) CD45RA(+) effector-memory CD8 T cells and CXCL10(+) macrophages are associated with response to atezolizumab plus bevacizumab in advanced hepatocellular carcinoma. Nat Commun. (2023) 1:7825. doi: 10.1038/s41467-023-43381-1, PMID: 38030622 PMC10687033

[89] Mikolajczyk TP Nosalski R Szczepaniak P Budzyn K Osmenda G Skiba D . Role of chemokine RANTES in the regulation of perivascular inflammation, T-cell accumulation, and vascular dysfunction in hypertension. FASEB J. (2016) 5:1987–99. doi: 10.1096/fj.201500088R, PMID: 26873938 PMC4836375

[90] Li Y Liu X Duan W Tian H Zhu G He H . Batf3-dependent CD8α(+) dendritic cells aggravates atherosclerosis via th1 cell induction and enhanced CCL5 expression in plaque macrophages. EBioMedicine. (2017) 18):188–98. doi: 10.1016/j.ebiom.2017.04.008, PMID: 28411140 PMC5405198

[91] Gruber T Kremenovic M Sadozai H Rombini N Baeriswyl L Maibach F . IL-32γ potentiates tumor immunity in melanoma. JCI Insight. (2020) 18:e138772. doi: 10.1172/jci.insight.138772, PMID: 32841222 PMC7526542

[92] Pan Y Xiong M Chen R Ma Y Corman C Maricos M . Athymic mice reveal a requirement for T-cell-microglia interactions in establishing a microenvironment supportive of Nf1 low-grade glioma growth. Genes Dev. (2018) 7-8:491–6. doi: 10.1101/gad.310797.117, PMID: 29632086 PMC5959233

[93] Zhou Z Zhou X Jiang X Yang B Lu X Fei Y . Single-cell profiling identifies IL1B(hi) macrophages associated with inflammation in PD-1 inhibitor-induced inflammatory arthritis. Nat Commun. (2024) 1:2107. doi: 10.1038/s41467-024-46195-x, PMID: 38453911 PMC10920757

[94] Luo F Li H Ma W Cao J Chen Q Lu F . The BCL-2 inhibitor APG-2575 resets tumor-associated macrophages toward the M1 phenotype, promoting a favorable response to anti-PD-1 therapy via NLRP3 activation. Cell Mol Immunol. (2024) 1:60–79. doi: 10.1038/s41423-023-01112-y, PMID: 38062129 PMC10757718

[95] Moser B . T-cell memory: the importance of chemokine-mediated cell attraction. Curr Biol. (2006) 13:R504–7. doi: 10.1016/j.cub.2006.06.008, PMID: 16824912

[96] Chu KL Batista NV Girard M Watts TH . Monocyte-derived cells in tissue-resident memory T cell formation. J Immunol. (2020) 3:477–85. doi: 10.4049/jimmunol.1901046, PMID: 31964721

[97] Iijima N Iwasaki A . T cell memory. A local macrophage chemokine network sustains protective tissue-resident memory CD4 T cells. Science. (2014) 6205:93–8. doi: 10.1126/science.1257530, PMID: 25170048 PMC4254703

[98] Garrido-Martin EM Mellows TWP Clarke J Ganesan AP Wood O Cazaly A . M1(hot) tumor-associated macrophages boost tissue-resident memory T cells infiltration and survival in human lung cancer. J Immunother Cancer. (2020) 2:e000778. doi: 10.1136/jitc-2020-000778, PMID: 32699181 PMC7375465

[99] Ren YF Ma Q Zeng X Huang CX Ren JL Li F . Single-cell RNA sequencing reveals immune microenvironment niche transitions during the invasive and metastatic processes of ground-glass nodules and part-solid nodules in lung adenocarcinoma. Mol Cancer. (2024) 1:263. doi: 10.1186/s12943-024-02177-7, PMID: 39580469 PMC11585206

[100] Moreno Ayala MA Campbell TF Zhang C Dahan N Bockman A Prakash V . CXCR3 expression in regulatory T cells drives interactions with type I dendritic cells in tumors to restrict CD8(+) T cell antitumor immunity. Immunity. (2023) 7:1613–30.e5. doi: 10.1016/j.immuni.2023.06.003, PMID: 37392735 PMC10752240

[101] Voskoboinik I Whisstock JC Trapani JA . Perforin and granzymes: function, dysfunction and human pathology. Nat Rev Immunol. (2015) 6:388–400. doi: 10.1038/nri3839, PMID: 25998963

[102] Farhood B Najafi M Mortezaee K . CD8(+) cytotoxic T lymphocytes in cancer immunotherapy: A review. J Cell Physiol. (2019) 6:8509–21. doi: 10.1002/jcp.27782, PMID: 30520029

[103] Li H Li J Bai Z Yan S Li J . Collagen-induced DDR1 upregulates CXCL5 to promote neutrophil extracellular traps formation and Treg infiltration in breast cancer. Int Immunopharmacol. (2023) 120):110235. doi: 10.1016/j.intimp.2023.110235, PMID: 37201403

[104] Chen S Saeed A Liu Q Jiang Q Xu H Xiao GG . Macrophages in immunoregulation and therapeutics. Signal Transduct Target Ther. (2023) 1:207. doi: 10.1038/s41392-023-01452-1, PMID: 37211559 PMC10200802

[105] Lei Q Zhen S Zhang L Zhao Q Yang L Zhang Y . A2AR-mediated CXCL5 upregulation on macrophages promotes NSCLC progression via NETosis. Cancer Immunol Immunother. (2024) 6:108. doi: 10.1007/s00262-024-03689-3, PMID: 38642131 PMC11032303

[106] Shiri AM Zhang T Bedke T Zazara DE Zhao L Lücke J . IL-10 dampens antitumor immunity and promotes liver metastasis via PD-L1 induction. J Hepatol. (2024) 4:634–44. doi: 10.1016/j.jhep.2023.12.015, PMID: 38160941 PMC10964083

[107] Khazaie K von Boehmer H . The impact of CD4+CD25+ Treg on tumor specific CD8+ T cell cytotoxicity and cancer. Semin Cancer Biol. (2006) 2:124–36. doi: 10.1016/j.semcancer.2005.11.006, PMID: 16443370

[108] Zheng Y Wang N Wang S Zhang J Yang B Wang Z . Chronic psychological stress promotes breast cancer pre-metastatic niche formation by mobilizing splenic MDSCs via TAM/CXCL1 signaling. J Exp Clin Cancer Res. (2023) 1:129. doi: 10.1186/s13046-023-02696-z, PMID: 37210553 PMC10199643

[109] Furudate S Fujimura T Kambayashi Y Kakizaki A Hidaka T Aiba S . Immunomodulatory effect of imiquimod through CCL22 produced by tumor-associated macrophages in B16F10 melanomas. Anticancer Res. (2017) 7:3461–71. doi: 10.21873/anticanres.11714, PMID: 28668835

[110] Xu W Wu Y Liu W Anwaier A Tian X Su J . Tumor-associated macrophage-derived chemokine CCL5 facilitates the progression and immunosuppressive tumor microenvironment of clear cell renal cell carcinoma. Int J Biol Sci. (2022) 13:4884–900. doi: 10.7150/ijbs.74647, PMID: 35982911 PMC9379407

[111] Zhao C Wang D Li Z Zhang Z Xu Y Liu J . IL8 derived from macrophages inhibits CD8(+) T-cell function by downregulating TIM3 expression through IL8-CXCR2 axis in patients with advanced colorectal cancer. Int Immunopharmacol. (2023) 121):110457. doi: 10.1016/j.intimp.2023.110457, PMID: 37331296

[112] de Masson A Darbord D Dobos G Boisson M Roelens M Ram-Wolff C . Macrophage-derived CXCL9 and CXCL11, T-cell skin homing, and disease control in mogamulizumab-treated CTCL patients. Blood. (2022) 12:1820–32. doi: 10.1182/blood.2021013341, PMID: 34905599

[113] Marcovecchio PM Thomas G Salek-Ardakani S . CXCL9-expressing tumor-associated macrophages: new players in the fight against cancer. J Immunother Cancer. (2021) 2:e002045. doi: 10.1136/jitc-2020-002045, PMID: 33637602 PMC7919587

[114] Luan X Lei T Fang J Liu X Fu H Li Y . Blockade of C5a receptor unleashes tumor-associated macrophage antitumor response and enhances CXCL9-dependent CD8(+) T cell activity. Mol Ther. (2024) 2:469–89. doi: 10.1016/j.ymthe.2023.12.010, PMID: 38098230 PMC10861991

[115] Freeman P Bellomo G Ireland L Abudula M Luckett T Oberst M . Inhibition of insulin-like growth factors increases production of CXCL9/10 by macrophages and fibroblasts and facilitates CD8(+) cytotoxic T cell recruitment to pancreatic tumours. Front Immunol. (2024) 15):1382538. doi: 10.3389/fimmu.2024.1382538, PMID: 39165364 PMC11334161

[116] He N Huang H Wu S Ji W Tai Y Gao R . Microwave ablation combined with PD-L1 blockade synergistically promotes Cxcl9-mediated antitumor immunity. Cancer Sci. (2024) 7:2196–208. doi: 10.1111/cas.16182, PMID: 38655660 PMC11247550

[117] Zhang J Liu J Chen H Wu W Li X Wu Y . Specific immunotherapy generates CD8(+) CD196(+) T cells to suppress lung cancer growth in mice. Immunol Res. (2016) 4:1033–40. doi: 10.1007/s12026-016-8793-y, PMID: 26910585

[118] Kumar S Mohan V Kant Singh R Kumar Gautam P Kumar S Shukla A . Tumor-derived Hsp70-CD14 interaction enhances the antitumor potential of cytotoxic T cells by activating tumor-associated macrophages to express CC chemokines and CD40 costimulatory molecules. Int Immunopharmacol. (2024) 138):112584. doi: 10.1016/j.intimp.2024.112584, PMID: 38944948

[119] Haabeth OA Lorvik KB Hammarström C Donaldson IM Haraldsen G Bogen B . Inflammation driven by tumour-specific Th1 cells protects against B-cell cancer. Nat Commun. (2011) 2):240. doi: 10.1038/ncomms1239, PMID: 21407206 PMC3072106

[120] Leung SY Yuen ST Chu KM Mathy JA Li R Chan AS . Expression profiling identifies chemokine (C-C motif) ligand 18 as an independent prognostic indicator in gastric cancer. Gastroenterology. (2004) 2:457–69. doi: 10.1053/j.gastro.2004.05.031, PMID: 15300578

[121] Yi M Li T Niu M Zhang H Wu Y Wu K . Targeting cytokine and chemokine signaling pathways for cancer therapy. Signal Transduct Target Ther. (2024) 1:176. doi: 10.1038/s41392-024-01868-3, PMID: 39034318 PMC11275440

